# Anti-inflammatory effects of fermented and non-fermented *Sophora flavescens*: a comparative study

**DOI:** 10.1186/1472-6882-11-100

**Published:** 2011-10-26

**Authors:** Chun-chao Han, Hong Wei, Jianyou Guo

**Affiliations:** 1 School of Pharnnacy, Shandong University of Traditional Chinese Medicine, Jinan 250355, People's Republic of China; 2 Shandong Medical College, Jinan 250002, People's Republic of China; 3 Key Laboratory of Mental Health, Institute of Psychology, Chinese Academy of Sciences, Beijing 100101, People's Republic of China

## Abstract

**Background:**

The roots of *Sophora flavescens *(Leguminosae) have been used in East Asian countries as an herbal medicine and a food ingredient for thousands of years. The aim of the present study was to investigate the effects of *S. flavescens *fermentation on endotoxin-induced uveitis (EIU) in rats.

**Methods:**

EIU was induced in rats via a footpad injection of lipopolysaccharide (LPS). Immediately after the LPS inoculation, fermented and non-fermented extracts of *S. flavescens *(FSE and NFSE, respectively) were administered orally, and the aqueous humor was collected from both eyes 24 hours later. The anti-inflammatory effects of FSE and NFSE were examined in terms of regulation of nuclear factor kappa B (NF-κB) activation and the expression of interleukin-1β (IL-1β), tumor necrosis factor-α (TNF-α), inducible nitric oxide synthase (iNOS), intercellular cell adhesion molecule (ICAM)-1, and cyclooxygenase-2 (COX-2). The regulation of maleic dialdehyde (MDA) levels and polymorphonuclear cell (PMN) infiltration by FSE and NFSE were also examined.

**Results:**

Treatment with FSE significantly inhibited LPS-induced increases in IL-1β and TNF-α production and the expression of iNOS, ICAM-1 and COX-2. Moreover, FSE suppressed LPS-induced NF-κB activation, and reduced both MDA levels and infiltration by PMN.

**Conclusion:**

These results indicate that solid state fermentation may enhance the anti-inflammatory effects of *S. flavescens*.

## Background

Inflammation plays an important role in a wide variety of chronic human diseases including cardiovascular diseases and cancer. Although various chemical regimens have been used to treat inflammation, efficacy can be unsatisfactory and may encounter the problem of drug resistance. To improve treatments based on anti-inflammatory chemicals, novel therapeutic strategies may be developed from medical plants used in Chinese Traditional Medicine. Of the huge array of candidates available, we chose to investigate *Sophora flavescens *(Leguminosae) because of its potential health promoting properties; it is anti-inflammatory, an anti-asthmatic, an anthelmintic, and a free radical scavenger, and it has antimicrobial activities and improves mental health [1-5].

The roots of *S. flavescens *(Leguminosae) have been traditionally used in East Asian countries as an herbal medicine and food ingredient for thousands of years. *S. flavescens *contains alkaloids, triterpenoids, and flavonoids, which are known to have various biological activities [6]. However, different processing methods (such as fermentation) change the properties of this material. Therefore, the aim of the present study was to develop a solid state fermentation (SSF) protocol for *S. flavescens *and to investigate whether this method led to an increase in its anti-inflammatory effects.

SSF is a fermentation process conducted on a solid support, which has low moisture content and occurs in a non-septic and natural state [7]. SSF is a cutting-edge, clean technology with great potential for use in the production or extraction of biologically active compounds from natural sources. Some studies show that SSF changes the properties of the plant materials; therefore, we compared the anti-inflammatory properties of fermented and non-fermented roots.

To better elucidate the anti-inflammatory effects of the fermented and non-fermented extracts of *S. flavescens *(FSE and NFSE, respectively), a rat model of endotoxin-induced uveitis (EIU) was created by inducing acute anterior segment intraocular inflammation via an injection of lipopolysaccharide (LPS) [8,9]. In this model, LPS directly stimulates the vascular endothelium, macrophages, and other inflammatory cells, which release factors such as nitric oxide [10,11], cytokines (including tumor necrosis factor (TNF-α)) [12], and eicosanoid mediators [13]. The levels of these molecules were measured in FSE- or NFSE-treated rat eyes. The results showed that SSF enhances the anti-inflammatory effects of *S. flavescens *in a rat model of EIU.

## Methods

### Plant material

The roots of *S. flavescens *were collected from Shandong Province, China, in October 2009. A fine powder for use in the extraction study was prepared by milling dry *S. flavescens *with a mechanical grinder and sieving through a 20-mesh metal sieve.

### Microorganisms and fermentation

The seeds of *Coprinus comatus *were purchased from the Agricultural Culture Collection of China. First, the seeds were grown at 28°C for 5 days on PDA slants (1,000 mL 20% potato extract liquid + 20.0 g dextrose + 20.0 g agar) and then maintained at 4°C in a refrigerator. Five to six pieces of *C. comatus *mycelia were transferred from a slant into 250 mL Erlenmeyer flasks containing 100 mL liquid medium (20% potato extract liquid + 2.0% dextrose + 0.1% KH_2_PO_4 _+ 0.05% MgSO_2_). The culture was incubated at 27°C on a rotary shaker at 180 rpm for 7 days [14,15].

Ground *S. flavescens *(50 g) was autoclaved in Erlenmeyer flasks (500 mL) for 20 min at 121°C. After cooling, the flasks were inoculated with 15 mL of inoculum and incubated at 27°C for 30 days.

### FSE and NFSE preparation

Fermented and non-fermented *S. flavescens *was dried at room temperature (24.2 ± 1.0°C), and refluxed and extracted three times for 4 h in boiling water at a dried material to solvent ratio of 1:6 (w/v). The supernatant was collected by filtration, and the solvent was evaporated under reduced pressure and lyophilized.

### Animals

Healthy male adult Wistar rats (2 months old and weighing 225 ± 25 g) were used in the study. The study was approved by Shandong University's ethics committee, and all procedures complied with the guidance set out in the Guidelines for Caring for Experimental Animals published by the Ministry of Science and Technology of the People's Republic of China. Every care was taken to minimize discomfort, distress, and pain.

EIU was induced by a footpad injection of 200 μg LPS (from *Escherichia coli*, Nanjing Duly Biotech Co., Ltd., China; 100 μg per footpad) diluted in 0.1 mL phosphate-buffered saline (pH 7.4).

Thirty EIU rats were selected and divided three equal groups: an endotoxin-treated group, an FSE-treated group, and an NFSE-treated group. The three groups were given oral saline, FSE (75 mg/kg/d) or NFSE (75 mg/kg/d), respectively. Ten normal control rats were given saline only. Twenty-four hours later, the rats were sacrificed and the aqueous humor (15-20 μL/rat) was collected from both eyes for measurement of inflammatory cells and inflammatory mediators.

### Measurement of maleic dialdehyde (MDA)

MDA, a reliable marker for lipid peroxidation, was measured using a thiobarbituric acid assay (Nanjing Jiancheng Bioengineering Institute) according to the manufacturer's instructions. The total protein content of the samples was measured using a Coomassie Blue assay (Nanjing Jiancheng Bioengineering Institute) and the MDA content (nmol/mg protein) calculated using the following formula: absorbance of sample tube/absorbance of standard tube × 2.5.

### Measurement of PMN infiltration

Myeloperoxidase (MPO) activity was measured to assess the extent of polymorphonuclear cell (PMN) infiltration into the eyes using a commercial assay kit (Nanjing Jiancheng Bioengineering Co., Ltd., China).

### Measurement of IL-1β and TNF-α

The concentration of interleukin-1β (IL-1β) and TNF-α in the aqueous humor was determined using a commercial ELISA kit (Shanghai Jinma Biological Technology Inc., China) according to the manufacturer's instructions.

### Measurement of NF-κB activation

Nuclear factor kappa B (NF-κB) activation in the eyes was determined using an ELISA kit (Shanghai Jinma Biological Technology Inc., China) according to the manufacturer's instructions. This kit specifically detects the p50 sub-unit of NF-κB.

### Measurement of ICAM-1, iNOS, and COX-2 levels

Expression of intercellular cell adhesion molecule (ICAM)-1, inducible nitric oxide synthase (iNOS), and cyclooxygenase-2 (COX-2) was examined using a commercially available immunohistochemistry kit (Hengdabaisheng Biotechnology, Beijing, China) according to the manufacturer's instructions.

### Statistical analysis

Data were expressed as the mean ± S.E.M. and the results analyzed by ANOVA followed by Dunnett's t test. A p value of < 0.05 was considered significant.

## Results and Discussion

*S. flavescens *has many pharmacological properties, including anti-inflammatory activity. Therefore, the aim of the present study was to investigate whether SSF enhanced the anti-inflammatory effects of *S. flavescens*.

During SSF, the microorganisms associated with fermentation degrade the plant cell walls, enabling the release of biologically active compounds during the next extraction stage. This results in high concentrations of biologically active compounds, which may enhance the anti-inflammatory effects of *S. flavescens*.

**Figure 1:**
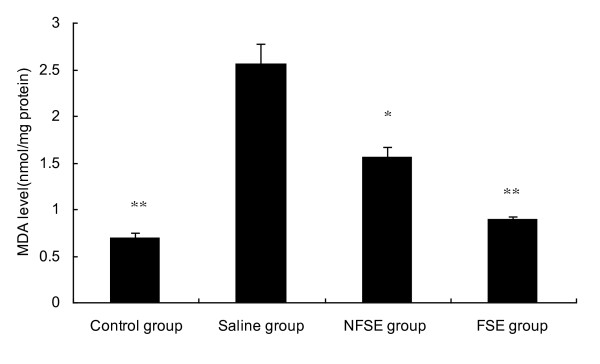
**Effects of FSE and NFSE on MDA levels**. Values represent the mean ± SEM. *p < 0.05 vs. saline group; **p < 0.01 vs. saline group.

Peroxidation damage plays an important role in the progression of LPS-induced injury; therefore, the anti-oxidant effects of FSE were investigated by measuring MDA levels. The control animals showed low MDA levels; however, the MDA levels in the saline group were significantly higher (p < 0.05). As shown in Figure 1, MDA level in the FSE and NFSE groups were significantly lower than those in the saline group (p < 0.01 and p < 0.05, respectively).

PMN infiltration is initiated by inflammatory mediators. In addition to direct neuronal damage, PMNs contribute to secondary injury by causing microvessel occlusion and releasing oxygen radicals, cytolytic proteases, and proinflammatory cytokines, which may injure the endothelium [16,17]. Chopp et al. reported that a reduction in the number of PMNs reduced post-ischemic tissue damage in the heart, intestine, lung, and liver [18]. The present study was undertaken to determine whether FSE reduces the number of PMNs in the aqueous humor. The activity of MPO was measured as an indicator of PMN migration. The results showed that MPO activity was relatively low in the control group, but significantly increased in the saline group. Treatment with FSE significantly reduced EIU-induced MPO activity compared with that in the saline group (p < 0.01, Table 1). NFSE treatment also reduced LPS-induced MPO activity; however, this was not statistically significant.

**Table 1:** Effects of FSE and NFSE on MPO activity

Groups	(U/g^-1^)
Control group	0.99 ± 0.21*
Saline group	2.08 ± 0.22
FSE group	1.11 ± 0.15*
NFSE group	1.87 ± 0.20

Values represent the mean ± SEM. *p < 0.05 vs. saline group.

**Figure 2:**
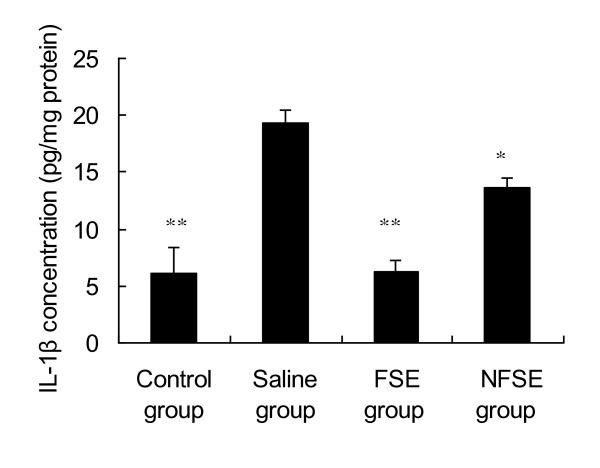
**Effects of FSE and NFSE on IL-1β concentration**. Values represent the mean ± SEM. *p < 0.05 vs. saline group; **p < 0.01 vs. saline group.

**Figure 3:**
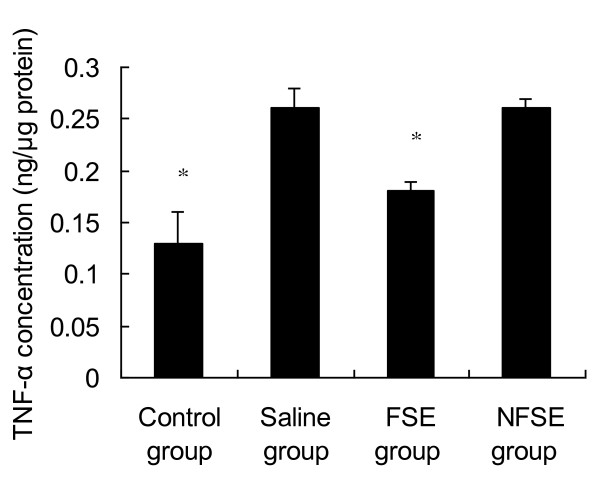
**Effects of FSE and NFSE on TNF-α concentration**. Values represent the mean ± SEM. *p < 0.05 vs. saline group.

Cytokines are small glycoproteins produced in response to an antigen, and were originally described as mediators for regulating the innate and adaptive immune responses. Thus, cytokine expression is upregulated in many diseases. Of these inflammatory mediators, IL-1β and TNF-α are of particular importance because they play a major role in coordinating the mechanisms that regulate pro-inflammatory responses [19]. Figure 2 shows that IL-1β levels were significantly increased in the aqueous humor of EIU rats. Treatment with both NFSE and FSE resulted in a marked decrease in IL-1β levels compared with those in the saline group (p < 0.05 and p < 0.01, respectively). In addition, the levels of TNF-α were significantly increased after endotoxin injection (Figure 3). FSE suppressed EIU-induced TNF-α production (p < 0.05); however, this was not the case in the NFSE-treated group.

**Figure 4:**
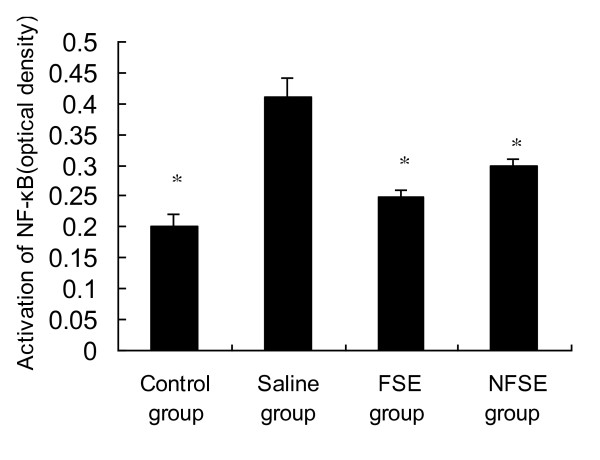
**Effects of FSE and NFSE on NF-κB activation**. Values represent the mean ± SEM. *p < 0.05 vs. saline group.

NF-κB comprises a family of transcription factors that regulate the expression of pro-inflammatory mediators [20]. NF-κB activation is correlated with significant increases in IL-1β and TNF-α mRNA levels [21]. Therefore, we hypothesized that FSE may potentially show beneficial effects by decreasing the expression of NF-κB. As shown in Figure 4, NF-κB expression in the saline group was significantly higher than that in the control group (p < 0.01), and FSE treatment significantly suppressed EIU-induced NF-κB expression. This is consistent with the results presented in Figure 2 and 3. Taken together, these results suggest that the inhibitory effects of FSE on expression of the NF-κB p50 subunit are associated with reduced concentrations of IL-1β and TNF-α in EIU rats.

As shown in Table 2, EIU rats expressed typical markers of inflammation, including upregulation of adhesion molecules and production of pro-oxidative enzymes (COX-2 and iNOS). The adhesion molecule, ICAM-1, has been extensively investigated. The expression of ICAM-1 in the EIU group was significantly higher than that in the control group. NFSE and FSE treatment markedly decreased the level of ICAM-1 expression compared with that in the saline group (p < 0.05 and p < 0.01, respectively).

**Table 2:** Effects of FSE and NFSE on ICAM-1, iNOS, and COX-2 protein production (number of immunopositive cells/mm^2^)

Groups	ICAM-1	iNOS	COX-2
Control group	22.22 ± 6.22**	10.41 ± 3.88**	9.06 ± 5.04**
Saline group	135.11 ± 21.35	68.28 ± 9.30	71.22 ± 8.23
FSE group	46.41 ± 1.23**	26.22 ± 1.39*	18.08 ± 3.22**
NFSE group	98.41 ± 33.31*	51.32 ± 2.59	50.11 ± 11.32*

Values represent the mean ± SEM. *p < 0.05 vs. saline group; **p < 0.01 vs. saline group.

Nitric oxide (NO) production, induced by bacterial LPS or cytokines, plays an important role in endotoxemia and inflammatory conditions [22], and selective inhibitors of iNOS inhibit the development of EIU [23]. Therefore, we wondered whether FSE, which inhibits NO production by inhibiting iNOS expression, had any positive therapeutic effects on inflammation. The results showed that FSE treatment inhibited the development of EIU and suppressed LPS-induced iNOS expression. Treatment with NFSE also reduced iNOS expression; however, this was not statistically significant.

NO also activates COX enzymes leading to a marked increase in PGE2 production [24]. COX-2 is primarily responsible for increased PGE2 production during inflammation, and PGE2 is generally considered to be a pro-inflammatory agent [25,26]. As shown in Table 2, the expression of COX-2 in the EIU group was significantly increased compared with that in the control group. FSE and NFSE treatment significantly decreased the expression of COX-2 protein in EIU rats (p < 0.01 and p < 0.05, respectively).

## Conclusion

The results of the present study clearly demonstrate the protective effects of FSE against EIU in rats. FSE treatment inhibited the production of EIU-induced inflammatory cytokines (IL-1β and TNF-α), decreased the levels of inflammation-related markers (MPO and ICAM-1), decreased the expression of pro-oxidative enzymes such as COX-2 and iNOS, and down-regulated the expression of NF-κB. Although both NFSE and FSE showed protective effects against EIU in rats, there was a significant difference between them. Taken together, the results of the present study show that SSF enhances the anti-inflammatory effects of *S. flavescens*.

## List of abbreviations

EIU: endotoxin-induced uveitis; LPS: lipopolysaccharide; FSE: fermented *S. flavescens *extract; NO: nitric oxide; NFSE: non-fermented *S. flavescens *extract; NF-κB: nuclear factor kappa B; MDA: maleic dialdehyde; PMN: polymorphonuclear cell; IL-1β: interleukin-1β; iNOS: inducible nitric oxide synthase; TNF-α: tumor necrosis factor-α; ICAM-1: intercellular cell adhesion molecule; COX-2: cyclooxygenase-2; SSF: solid state fermentation.

## Competing interests

The authors declare that they have no competing interests.

## Authors' contributions

CH designed this study and performed the laboratory analyses and statistical analysis. JG drafted the manuscript along with the other authors. All authors have read and approved the final manuscript.

## Pre-publication history

The pre-publication history for this paper can be accessed here:

http://www.biomedcentral.com/1472-6882/11/100/prepub
